# Pressure Ulcers in Admitted Patients at a Tertiary Care Hospital

**DOI:** 10.7759/cureus.24298

**Published:** 2022-04-20

**Authors:** Mustafa Qazi, Almas F Khattak, Muhammad T Barki

**Affiliations:** 1 Medicine and Surgery, Northwest School of Medicine, Peshawar, PAK; 2 Community Medicine and Research, Northwest School of Medicine, Peshawar, PAK; 3 Neurological Surgery, North West General Hospital and Research Center, Peshawar, PAK

**Keywords:** patient safety improvement, admitted patients, pakistan, bed sores, covid-19, survey, retrospective study, pressure ulcer, prevalence

## Abstract

Background

Pressure ulcers (PUs) occur when the skin covering a weight-bearing part of the body is compressed for a long time between bone, any other part of the body, bed, chair, or any other hard surface. This study aimed to determine the prevalence of pressure ulcers at a tertiary care hospital in all specialty departments including COVID-19.

Methods

A retrospective study was conducted at North West General Hospital and Research Center, Peshawar, Pakistan. After obtaining ethical approval, data were obtained from the hospital database from July 2020 to June 2021. The keywords “bed sore,” “pressure sore,” and “pressure ulcer” were used to search for relevant cases, and patient demographics, including age and gender, site of pressure ulcer, stage of pressure ulcer, whether the pressure ulcer was single or multiple, length of stay at the hospital, and specialty department, were collected.

Results

In total, 99 patients met the inclusion criteria, of which 65 (65.7%) were males, while 34 (34.3%) were females. The age of the patients ranged from 15 to 92 years, with a mean age of 59.93 years. Of the patients, 87 (87.9%) had acquired only a single pressure ulcer. Stage 2 pressure ulcers were the most documented, making up 43.1% of the total cases reported, while stage 4 cases were only 3.3%. The sites most frequently affected by pressure ulcers were the gluteal and sacral regions, accounting for 34.4% and 30.3%, respectively. The incidence of pressure ulcers was the highest in the COVID-19 ward, i.e., 25.3%, followed by the neurosurgery ward with a 20.2% incidence.

Conclusion

Pressure ulcers occur frequently in almost all the specialty departments of a healthcare setting, especially in COVID-19 and neurosurgery wards, and impose significant physical, psychological, and financial burdens. The prevention of pressure ulcers is the best approach to avert patients and their families from all the burdens associated with pressure ulcers.

## Introduction

Pressure ulcers (PUs) previously known as decubitus ulcers and informally called bedsores are caused by intense and/or prolonged pressure or in combination with shear usually in areas of the body where there are bony prominences [1]. They occur when the skin covering a weight-bearing part of the body is compressed for a long time between bone, any other hard surface, or a medical device such as oxygen tubing behind ears and endotracheal tubes. It is known that PUs are likely to occur in the aged, seriously ill, neurologically compromised or those with deficient nutrition [2-6]. Other factors such as race and socioeconomic status have also been documented [7,8].

The incidence of PUs in the United States and Canada is between 5% and 32% [9]. In the last few decades, many manuscripts have been published on PUs, including large-scale epidemiological surveys, but most have been done in first-world countries. On the contrary, studies in Pakistan have focused on PUs in patients with neurotrauma and spinal cord injury, ignoring other specialty departments [10,11]. This leads us to the conclusion that although PUs frequently occur in our country, there is poor statistical data to estimate their detrimental effects or the financial burden on patient health and safety.

Evidence-based studies to prevent and treat PUs have led us to design guidebooks, but unfortunately, they are not adequately used in clinical practices [12]. One reason may be that we are failing to educate our healthcare workers, patients, their family members, and our community about pressure ulcer (PU) care. However, even if PU care is a multidisciplinary responsibility, nurses play a major role; thus, preventing ulcers should be the goal of all nursing staff.

This study is part of a patient safety and quality improvement project by the authors that compares the prevalence of pressure ulcers pre- and post-intervention using a pressure ulcer prevention tool from the Agency for Healthcare Research and Quality [13]. The project has also been selected for ePoster presentation at the International Forum on Quality and Safety in Healthcare Gothenburg 2022. The aim of this study is to determine the frequency of PUs at a tertiary care hospital in all specialty departments including the COVID-19 ward.

## Materials and methods

Study setting and design

A retrospective survey was conducted at North West General Hospital and Research Center, Peshawar, Pakistan, to determine and compare the occurrence of pressure ulcers in the different specialty wards at the facility. The facility is a private sector tertiary care hospital that caters to the healthcare needs of more than 1000 patients daily. Patients are received from all over Pakistan, especially from the Khyber Pakhtunkhwa region and Afghanistan.

Ethical approval

Ethical approval for the study was granted by the Institutional Review and Ethics Board of North West General Hospital and Research Center after approval of the project proposal. The approval is registered under reference ID IREB/EAC/04 in the institution records.

Inclusion and exclusion criteria

Those patients who were admitted to the facility in the past one year for at least three days and had acquired pressure ulcers during their stay (hospital-acquired pressure ulcers (HAPUs)) were included in the study. Patients with incomplete data were excluded from the study.

Data collection and analysis

Data were collected from the hospital database of the patients’ physicians and nursing notes at the Management and Information Section (MIS) of the facility. The keywords “bed sore,” “pressure sore,” and “pressure ulcer” were used to search for relevant cases, and patient demographics, including age and gender, site of PU, stage of PU, whether the PU was single or multiple, length of stay at the hospital, and specialty department, were collected. The data of 18,579 patients from July 2020 to June 2021 were obtained, of which 99 patients were identified who met the inclusion criteria. Eight such patients who were admitted to the Accident and Emergency department with existing PUs were excluded from the study. Multiple PUs were counted as separate cases in the stage and anatomical location variables only. Data were exported to SPSS version 21 (IBM Corporation, Armonk, NY, USA), analyzed, and formatted into tables.

## Results

After retrieving and reviewing the data collected, 99 patients were identified who met the inclusion criteria. Regarding gender, 65 (65.7%) patients were males, while 34 (34.3%) were females. The age of the patients ranged from 15 to 92 years, with a mean age of 59.93 years. Six (6%) patients were under the age of 30, 22 (22.2%) were between 30 and 50 years of age, the highest number of patients (43 (43.4%)) were in the age group of 51-70 years, and 28 (28.2%) were older than 70 years.

A total of 122 cases of hospital-acquired pressure ulcers (HAPUs) were reported. Of the patients, 87 (87.9%) had acquired only a single PU. Stage 2 PUs were the most documented, making up 43.1% of the total cases reported, while stage 4 cases were only 3.3%. The sites most frequently affected by HAPUs, identified and documented by the attending physicians and nurses, were the gluteal and sacral regions, accounting for 34.4% and 30.3%, respectively. Conversely, the elbows and trochanters were the least affected of all the areas of the body, which were six cases (4.9%) each (Table 1).

**Table 1:** Details of pressure ulcers

Detail	Number (%)	
Single/multiple		
Single	87 (87.9)	
Multiple	12 (12.1)	
Stage		
Stage 1	37 (30.1)	
Stage 2	53 (43.1)	
Stage 3	15 (12.2)	
Stage 4	4 (3.3)	
Anatomical location		
Gluteal	42 (34.4)	
Sacrum	37 (30.3)	
Scapular	14 (11.4)	
Heels	10 (8.2)	
Elbows	6 (4.9)	
Ischium	7 (5.7)	
Trochanters	6 (4.9)	

The length of stay had an indirect relation to the number of patients. Thirty-three (33.3%) patients stayed at the hospital for less than 10 days; on the contrary, only 12 (12.1%) patients had a stay of longer than 40 days. The incidence of PUs was the highest in the COVID-19 ward, i.e., 25.3%, followed by the neurosurgery ward with 20.2% incidence. In contrast, the orthopedic ward had only one case of PU reported (Table 2).

**Table 2:** Patients’ stay at the hospital

Detail	Number (%)
Length of stay	
<10 days	33 (33.3)
10-20 days	27 (27.3)
21-40 days	27 (27.3)
>40 days	12 (12.1)
Department	
Cardiology	6 (6.1)
COVID-19	25 (25.3)
Endocrinology	3 (3)
Gastroenterology	2 (2)
General medicine	12 (12.1)
General surgery	5 (5.1)
Gynecology	2 (2)
Nephrology	8 (8.1)
Neurosurgery	20 (20.2)
Orthopedic	1 (1)
Pulmonology	15 (15.2)

## Discussion

This study reveals that PUs were more likely to affect only one location of a patient’s body, which may be attributed to the fact that multiple injuries are easily noticeable by the healthcare professionals on duty. The same can be said for the stage of PUs, in which, as shown in Table 1, the number of patients regularly decreases from stage 1 to stage 4 because consistent inspections of the patient’s skin integrity lead to early discovery and treatment of PUs. However, if the early signs of damaged skin are left untreated, they lead to extensive destruction and tissue necrosis known as stage 4 PUs.

In patients who are bound to wheelchairs, the gluteal and sacral regions are more prone to PUs since they are the only area under pressure. The results of our study regarding the most affected anatomical location by PUs are in accordance with other studies conducted [14,15]. The elbows and trochanters were the least reported sites to have acquired PUs. Contrary to this study, heels have also been reported as the most common site [15,16]. People with disabilities usually have catheters or other devices inserted for procedures during their stay, which may account for them being the least affected by PUs; however, further studies need to be conducted to determine why some areas of the body are more prone to PUs than others. The number of cases decreased with the increase in length of stay, which is interesting as no reason can be brainstormed to back up the trend. Less number of patients who stayed for more than 40 days were included in this study. Further studies should be conducted on these patients after they are discharged and shifted to long-term care facilities to correlate the length of stay and the incidence of HAPUs. Our findings are in contrast to studies previously conducted [17,18]. Most of the cases were in patients who stayed for less than 10 days at the facility.

A quarter of the total cases reported were in the COVID-19 ward, but since no studies have been previously conducted on PU prevalence in patients with COVID-19, it is not possible to provide a comparison to the literature. It is however an alarming situation since patients with COVID-19 and their families already suffer a lot, and PU incidence further worsens their conditions. The lack of personal protective equipment (PPE) in the facility and the shortage of staff in the COVID-19 ward may also have added to the incidence of PUs. The neurosurgery ward had the second most reported cases of PUs, which supports the findings of studies previously conducted on patients with spinal cord injury and other neurotrauma [19,20]. The least reported cases were in the orthopedic, gynecology, and gastroenterology wards since patients do not have mobility issues in most conditions.

PUs remain a problem for all healthcare facilities, and because they can occur during short stays, prevention is very important. Studies show that reduced activity is the most significant risk factor for PUs [21,22]. Other factors that have been reported in past studies include shearing, nutritional deficiency, stool and urinary incontinence, smoking, anemia, and no use of pressure redistribution support surfaces [23-28].

Specific risk factors need to be determined in prospective studies. Rather than focusing on the treatment of PUs, which costs huge amounts, preventive measures should be encouraged and taught in all healthcare settings and the community. Certified wound care nurses should be hired or trained, who would implement the already introduced policies and pressure ulcer prevention protocols of the facility. PU care plans should be developed, and immobilized patients should be regularly assessed for the risk factors of PUs. The Wound, Ostomy, and Continence Nurses Society (WOCN) recommends minimizing/eliminating pressure, friction, and shear, regular positioning, prophylactic dressings in at-risk patients, and using incontinence skin barriers such as creams, ointments, and film-forming protectants in addition to optimum nutrition [29].

This study has not taken into account the deep tissue pressure injuries of patients with COVID-19 that mostly occur while the patients are in the prone position. This is a limitation of the study, and prospective studies need to be conducted that will consider the aforementioned aspect of hospital-acquired pressure ulcers.

## Conclusions

This study on the prevalence of PUs leads us to the conclusion that PUs occur in all specialty departments of a healthcare setting, especially in COVID-19 and neurosurgery wards. PUs impose significant physical, psychological, and financial burdens not only on patients but also on their relatives and caregivers. Given the current evidence, the prevention of pressure ulcers by methods including repositioning, optimizing the nutrition, and moisturizing the skin seems the most appropriate approach to prevent patients and their families from all the burdens associated with pressure ulcers.
